# lncRNA TCONS_00251376 Promotes the Proliferation and Migration of Gastric Cancer Cell Through Upregulating ETV1

**DOI:** 10.1002/cai2.156

**Published:** 2024-12-12

**Authors:** Dengfeng Ren, Fuxing Zhao, Jinming Li, Xinjian Guo, Xinfu Ma, Yonghui Zheng, Guoshuang Shen, Jiuda Zhao

**Affiliations:** ^1^ Breast Disease Diagnosis and Treatment Center, Affiliated Hospital of Qinghai University, Affiliated Cancer Hospital of Qinghai University Xining China; ^2^ Qinghai Provincial Clinical Research Center for Cancer; Qinghai Provincial Institute of Cancer Research Xining China; ^3^ Graduate School, Qinghai University Xining China; ^4^ Department of Pathology Affiliated Hospital of Qinghai University, Affiliated Cancer Hospital of Qinghai University Xining China; ^5^ Department of Gastrointestinal Oncology Surgery Affiliated Hospital of Qinghai University, Affiliated Cancer Hospital of Qinghai University Xining China

**Keywords:** ETV1, gastric cancer, gene, long noncoding RNA

## Abstract

**Background:**

Although there have been significant advancements in the treatment modalities for gastric cancer (GC) in recent years, the overall prognosis remains poor, particularly for individuals in advanced stages. The absence of a sensitive tumor marker in GC is a crucial factor contributing to this challenge.

**Methods:**

Our study focused on investigating a newly discovered long noncoding RNA (lncRNA) known as TCONS_00251376, which has been confirmed to exhibit differential expression in GC compared to adjacent tissues. To further validate these expression differences, we collected 22 pairs of GC and adjacent noncancerous tissues. Subsequent cell function experiments and animal studies were conducted to elucidate the role and underlying mechanisms of lncRNA TCONS_00251376 in the development of GC.

**Results:**

The study revealed a significant upregulation of lncRNA TCONS_00251376 in cancer tissues (*p *< 0.01) and a consistent upregulation in GC cell lines (AGS, MKN45, BGC‐823, and MGC‐803). Furthermore, it was observed that lncRNA TCONS_00251376 played a promotive role in the proliferation, migration, and invasion of GC cells. Subsequent analysis indicated that lncRNA TCONS_00251376 could upregulate the expression of ETV1, a factor associated with the prognosis of GC.

**Conclusions:**

Therefore, our findings suggest that lncRNA TCONS_00251376 functions as an oncogenic lncRNA, promoting tumorigenesis and progression by regulating the expression of ETV1 gene. This highlights its potential as an effective target for treating GC.

## Graphical abstract

The study revealed a significant upregulation of LncRNA TCONS_00251376 in cancer tissues and a consistent upregulation in GC cell lines (AGS, MKN45, BGC‐823, and MGC‐803). It was observed that LncRNA TCONS_00251376 played a promotive role in the proliferation, migration, and invasion of GC cells. Also, the study indicated that LncRNA TCONS_00251376 could upregulate the expression of ETV1, a factor associated with the prognosis of GC. Our findings suggest that LncRNA TCONS_00251376 functions as an oncogenic LncRNA, promoting tumorigenesis and progression by regulating the expression of ETV1 gene. This highlights its potential as an effective target for treating GC.

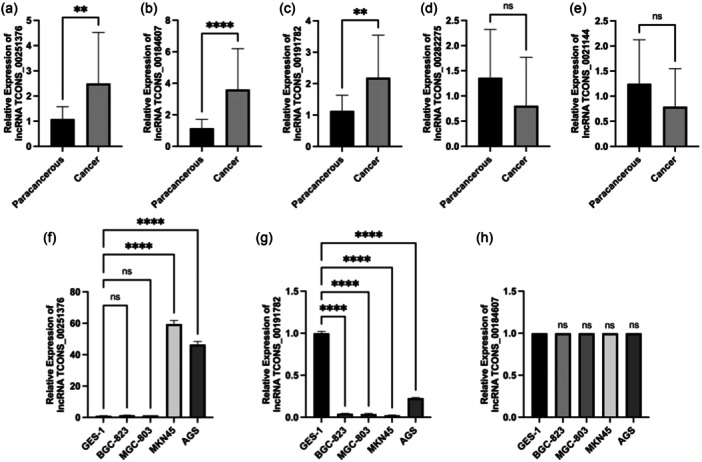

**Abbreviations**
ASOs: antisense oligonucleotides
FBS: fetal bovine serum
GC: gastric cancer
HRP: horseradish peroxidase
IARC: The International Agency for Research on Cancer
LncRNA: long noncoding RNA
MAPK: mitogen‐activated protein kinase
ncRNAs: noncoding RNAs
NSCLC: non‐small cell lung cancer
PBS: phosphate‐buffered saline
PI: propidium iodide
qRT‐PCR: real‐time quantitative PCR
RPMI: Roswell Park Memorial Institute
SDS‐PAGE: sodium dodecyl sulfate‐polyacrylamide gel electrophoresis

## 1 Introduction

Gastric cancer (GC) ranks fifth globally in terms of incidence and third in mortality [1]. According to the International Agency for Research on Cancer (IARC), China reported around 679,100 new cases and 498,000 deaths in 2015, constituting 42.5% of global cases, making it one of the regions with the highest incidence and mortality [2]. Unfortunately, over 80% of GC cases in China are diagnosed at an advanced stage, leading to a bleak prognosis [3]. Despite advancements in treatment options like surgery, chemotherapy, targeted therapy, immunotherapy, and radiotherapy, the overall prognosis for GC remains poor. This is particularly true for patients in advanced stages who miss the opportunity for radical surgery [4, 5].

The unfavorable prognosis of GC stems from the low rate of early‐stage diagnoses and suboptimal treatment outcomes. A significant factor contributing to the challenge of early detection is the absence of sensitive tumor markers for GC. Furthermore, the lack of molecular markers that can precisely guide molecular typing and personalized treatment for advanced‐stage GC patients results in unsatisfactory outcomes for this cohort. Despite numerous studies on the pathogenesis of GC and efforts to employ molecular markers for clinical diagnosis and treatment guidance, the understanding of its pathogenesis remains unclear. Unlike certain tumors such as non‐small cell lung cancer (NSCLC) and breast cancer, where multiple markers like EGFR, K‐RAS, T790M (for NSCLC), and ER, PR, HER‐2, Ki67 (for breast cancer) aid in molecular typing and guide individualized diagnosis and treatment, GC has only successfully utilized one molecular marker, HER‐2, for personalized treatment [5, 6].

In this study, we assessed the expression levels of a novel long noncoding RNA (lncRNA), specifically TCONS_00251376, in both GC tissues and adjacent paracarcinoma tissues. Additionally, we explored the impact of lncRNA TCONS_00251376 expression on the proliferation, migration, and invasion of GC cells.

## 2 Methods

### 2.1 Screening of New lncRNAs

Cancer tissues and adjacent noncancerous tissues were obtained from three patients who underwent gastric surgery at the Affiliated Hospital of Qinghai University. These tissue samples were then sent to Guangzhou Ribo Biotechnology Co., Ltd. for high‐throughput RNA sequencing. Based on expression abundance, log_2_ fold change values, and *p* values, three upregulated and two downregulated lncRNAs in cancer tissues were selected from the unnamed differentially expressed lncRNAs identified through high‐throughput RNA‐seq. These selected lncRNAs will undergo subsequent verification experiments.

### 2.2 Cell Lines and Cell Culture

We bought human GC cell lines, including BGC‐823, MGC‐803, AGS, and MKN45, as well as the normal human gastric mucosal epithelial cell line GES‐1 from Shanghai Zhong Qiao Xin Zhou Biotechnology Co., Ltd. These cell lines are cultured in Roswell Park Memorial Institute (RPMI) 1640 medium supplemented with 10% fetal bovine serum (FBS), 100 U/mL penicillin, and 100 μg/mL streptomycin. The cultivation takes place in a humidified incubator with 5%CO_2_ at 37°C.

### 2.3 Clinical Sample

Twenty‐two paired primary GC tissues and adjacent noncancerous tissues were collected from patients diagnosed with GC based on histopathologic evaluation. These patients underwent surgery at the Affiliated Hospital of Qinghai University between April 2019 and July 2020. Those who had undergone chemotherapy or radiotherapy before surgery were excluded from the study. Immediately after resection, all tissue samples were frozen in liquid nitrogen and stored at −80°C for subsequent analysis. The research protocol received approval from the ethical committee of the Affiliated Hospital of Qinghai University, and informed consent was obtained from all patients before their inclusion in the study.

### 2.4 Cell Transfection

Our previous studies have revealed that lncRNA TCONS_00251376 predominantly localizes in the nucleus. In efforts to modulate nuclear‐localized lncRNAs more effectively, we employed antisense oligonucleotides (ASOs), known to be more efficient than siRNAs for this purpose [7]. For ASO transfection, cells were seeded at 2 × 10^4^ per well in a 24‐well plate and transfected with specific ASOs (100 nM, RiboBio) following the manufacturer's instructions. The sequence of the ASOs used is as follows: ‐TGAGGCTAAAGCACAAGCCA‐.

### 2.5 RNA Extraction, Reverse Transcription, and Quantitative Real‐Time Polymerase Chain Reaction (qRT‐PCR)

Total RNA was extracted from GC tissues and cells using TRIzol reagent (Invitrogen) following the manufacturer's instructions. The NanoDrop 2000 (ThermoFishe) was utilized for quantifying RNA concentrations. cDNA synthesis was performed using the TransScript‐Uni One‐Step gDNA Removal and cDNA Synthesis SuperMix Kit (TransGen Biotech) according to the provided instructions. qRT‐PCR with SYBR Green dye (Takara) was employed to assess the expression levels of lncRNA TCONS_00251376 and other genes. The LightCycler 480 II system (Roche) was used for qRT‐PCR. GAPDH served as the internal control, and the relative expressions were normalized to GAPDH using the 2^−ΔΔCt^ method.

### 2.6 Cell Proliferation

Cell proliferation ability was assessed through a cell counting kit‐8 (CCK‐8) assay from Boster (Wuhan, China). Cells were seeded in 96‐well plates at a density of 5 × 10^3^ cells/well with a volume of 100 uL and cultured in RPMI‐1640 DMEM with 10% FBS for 24 h. Subsequently, 10 uL of CCK‐8 solution was added to each well, and the plates were incubated in a humidified atmosphere with 5%CO_2_ at 37°C for 1 h. Absorbance values were measured at a wavelength of 490 nm using the Infinite 200 PRO microplate reader (TECAN).

### 2.7 Cell Migration and Invasion

A wound‐healing assay was employed to assess cell migration capabilities. Cells treated with different conditions (3 × 10^5^ cells each) were individually seeded in 6‐well plates and cultured for 12–24 h. When the cultures reached 75% confluency, a sterile plastic tip was used to create a scratch in the cell layer. The plates were then washed with culture medium without FBS and cultured for an additional 24 h. Images of the plates were captured using a microscope (Olympus). Image J software was utilized to quantify the relative areas of the wounds.

Additionally, cell migration was evaluated using Transwell chambers with 8.0 µm Pore Polyester Membrane Inserts (Corning). A total of 100 µL FBS‐free medium containing 3 × 10^5^ cells from each group was added to the upper chamber, while 600 µL RPMI‐1640 DMEM with 10% FBS was added to the lower chamber. The cells in the chamber were incubated for 24 h in a humidified atmosphere with 5%CO_2_ at 37°C. Subsequently, cotton swabs were used to remove cells from the upper surface of the membrane. The cells that had migrated to the bottom of the membrane were fixed with a 4% formaldehyde solution and stained with 0.1% Crystal Violet Staining Solution. The migrated cells on the bottom of the membrane were then counted using a microscope (Olympus).

For the cell invasion assay, Transwell chambers with Matrigel‐coated upper chambers (Corning) were employed. A total of 100 µL FBS‐free medium containing 3 × 10^5^ cells from each group was added to the upper chamber, while 600 µL RPMI‐1640 DMEM with 10% FBS was added to the lower chamber. The cells in the chamber were incubated for 48 h in a 5%CO_2_ atmosphere at 37°C. After incubation, cells on the upper surface of the membrane were carefully scraped off. The cells that had invaded through the Matrigel to the bottom of the membrane were fixed, stained, and counted using a microscope (Olympus).

### 2.8 Cell Apoptosis and Cycle Analysis

Cells were stained with Annexin V and propidium iodide using the Annexin V–FITC Apoptosis Detection kit (Invitrogen), and the percentage of apoptosis was assessed using flow cytometry (BD Bioscience). For the detection of cell cycle, cells were stained with propidium iodide (PI) after 48 h of transfection, and analysis was performed using the FACS Calibur system (Beckman Coulter). To evaluate the number of apoptotic cells, Annexin V staining was carried out using FITC‐labeled Annexin V antibody and PI (BioLegend). Subsequently, the stained cells were analyzed using flow cytometry, and the results were analyzed using Flowjo software (Ashland).

### 2.9 Associated Target Gene Search of lncRNA TCONS_00251376

Input the sequence of lncRNA TCONS_00251376 into the Ensembl database (https://www.ensembl.org) to find the genes located upstream and downstream of lncRNA TCONS_00251376. Then we searched the literature to screen for genes related to GC.

### 2.10 Western Blot Analysis

Western Blot Experiment is an experiment in which electrophoretically separated components are transferred from the gel to a solid phase support (NC membrane or PVDF membrane) and detected with a specific reagent for an acid sequence of the amino acid as a probe. Protein extraction from AGS cells was carried out using RIPA lysis buffer containing protease and phosphatase inhibitors (Boster). The extracted proteins were then separated by sodium dodecyl sulfate‐polyacrylamide gel electrophoresis (SDS‐PAGE) and transferred to polyvinylidene difluoride membranes. Subsequently, the membranes were incubated overnight at 4°C with primary antibodies against β‐actin and ETV1. Following the primary antibody incubation, anti‐mouse or rabbit horseradish peroxidase (HRP)‐conjugated secondary antibodies were applied, and the membranes were further incubated at room temperature for 1 h. The protein–antibody complex was visualized using the ChemiScope mini assay.

### 2.11 Animal Experiment

A 150 μL AGS cell suspension with a concentration of 2 × 10^6^/mL was subcutaneously injected into the right forelimb armpit of 4–6‐week‐old BALB/c female mice weighing 16–18 g, with six mice per group. Tumor growth and the weight of mice were monitored and recorded every 4 days post‐transplantation using calipers and a digital scale. Once the tumor volume of all mice reached 100 mm^3^, multiple intratumoral injections were administered in phosphate‐buffered saline (PBS) buffer solution containing ASO‐lnc‐TCONS_00251376 and ASO‐NC (5 nmol per injection, RiboBio) every 3 days for a duration of 4 weeks. At the conclusion of the experiment, all mice were euthanized, and the weight and volume of the tumors were measured.

### 2.12 Statistical Analysis

For continuous variables, the results were reported as mean ± SD. Statistical significance of differences between two groups was determined using Student's *t*‐test. Correlation analysis was conducted using the chi‐square test. A two‐sided *p* < 0.05 was considered statistically significant. All statistical analyses were conducted using SPSS version 19 software (SPSS).

## 3 RESULTS

### 3.1 Screening of Newly Discovered Differentially Expressed lncRNAs

A total of 69 unnamed lncRNAs exhibiting differential expression in cancer tissues and paracancerous tissues were identified through RNA next‐generation sequencing, based on a log_2_ fold change >2 criterion. Among these, 44 lncRNAs were upregulated in cancer tissues, while 25 were downregulated. Subsequently, we selected three upregulated lncRNAs (lncRNA TCONS_00251376, lncRNA TCONS_00191782, and lncRNA TCONS_00184607) and two downregulated lncRNAs (lncRNA TCONS_00021144 and lncRNA TCONS_00282275) in cancer tissues, guided by the expression abundance of lncRNAs in both cancer tissues and adjacent tissues, for further investigation.

### 3.2 lncRNA TCONS_00251376 is Upregulated in GC Tissues

A total of 22 GC patients' cancer tissue and paracancerous tissue samples were collected. The majority of the patients were elderly, constituting 45.5% of the sample. Male patients represented 81.8% of the cohort. In terms of tumor characteristics, 54.5% of the patients had a tumor diameter ≥ 3.5 cm, and 86.4% of the lesions were located in the stomach, with 13.6% located in the cardia. A significant proportion of patients (63.6%) were in the III–IV phase, indicating advanced stages. Lymph node metastasis was observed in 81.8% of the patients, and 77.3% of the patients had T3/T4 stage, while 59.1% had N2/N3 stage. Regarding the pathological Lauren classification of the 22 patients, the intestinal type accounted for 40.9%, the diffuse type accounted for 36.4%, and the mixed type accounted for 22.7%, as illustrated in Table 1.

**Table 1:** Clinicopathological characteristics of 22 patients with gastric cancer.

Characteristics	Number (%)
Age (years)	
< 60	12 (54.5)
≥ 60	10 (45.5)
Gender	
Male	18 (81.8)
Female	4 (18.2)
Tumor size (cm)	
< 3.5	10 (45.5)
≥ 3.5	12 (54.5)
Primary tumor site	
Gastric	19 (86.4)
Gastroesophageal junction	3 (13.6)
Clinical staging	
I–II	8 (36.4)
III–IV	14 (63.6)
Lymphatic metastasis	
Yes	18 (81.8)
No	4 (18.2)
T stage	
T1/T2	5 (22.7)
T3/T4	17 (77.3)
N stage	
N0/N1	9 (40.9)
N2/N3	13 (59.1)
Lauren classification	
Intestinal type	9 (40.9)
Diffuse type	8 (36.4)
Mixed type	5 (22.7)

**Figure 1:**
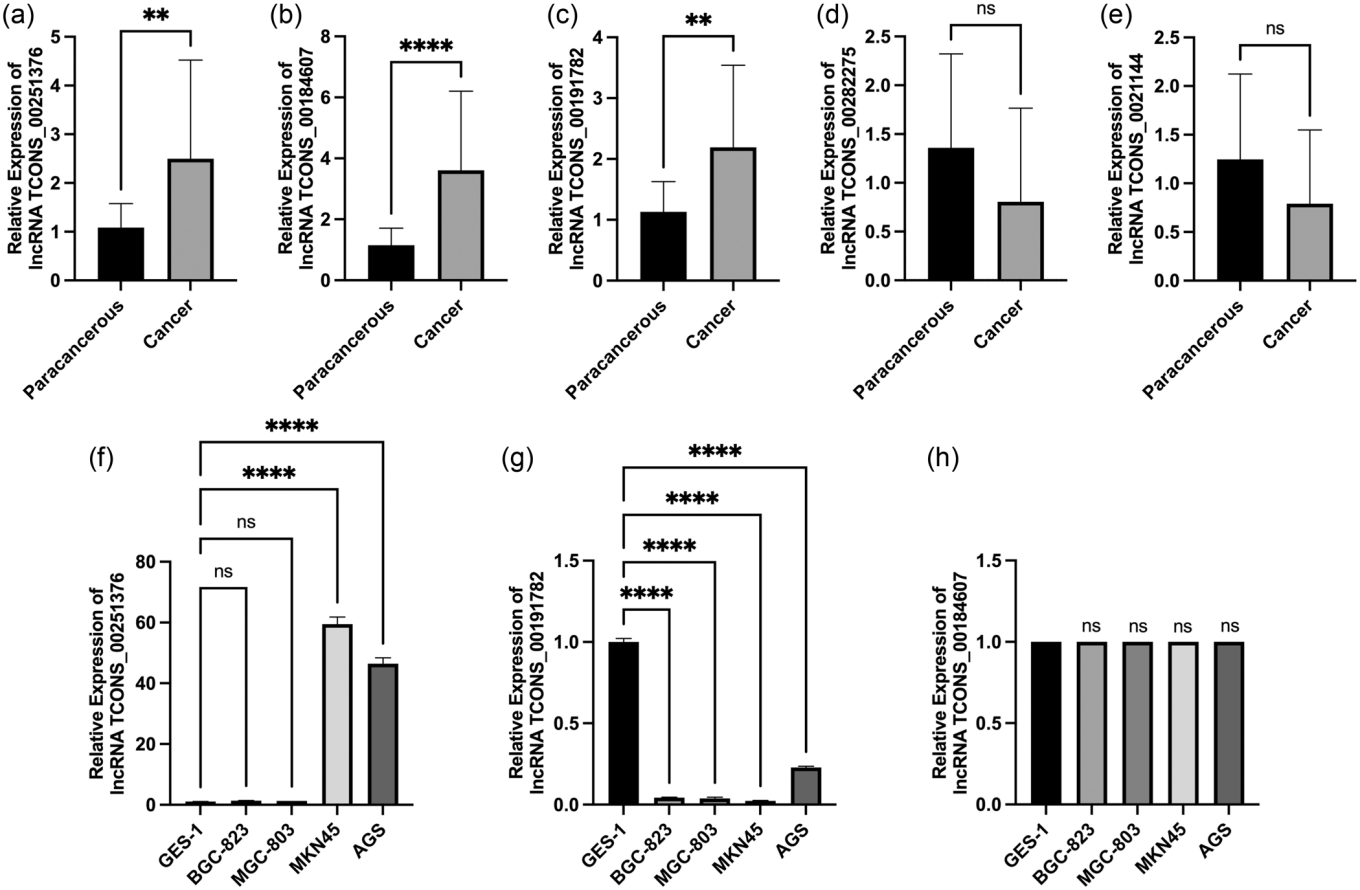
The expression of different lncRNAs in tissues and different gastric cancer cells. (a–e) The expression of lncRNA TCONS_00251376, lncRNA TCONS_00184607, lncRNA TCONS_00191782, lncRNA TCONS_00282275, and lncRNA TCONS_00021144 in tumor tissue and paracancer tissue. (f–h) The expression of lncRNA TCONS 00251376, lncRNA TCONS_00191782, and lncRNA TCONS_00184607 in five GC cells: GES‐1, BGC‐823, MGC‐803, MKN45, and AGS. **p < 0.01, ****p < 0.0001, ns: not significant.

To ascertain whether the expression of five unnamed lncRNAs in GC tissues significantly differed from that in adjacent tissues, we conducted qRT‐PCR to measure the expression levels of these lncRNAs in 22 pairs of GC tissues and adjacent tissues. The results revealed that among the five lncRNAs examined, three lncRNAs (lncRNA TCONS_00251376, lncRNA TCONS_00191782, and lncRNA TCONS_00184607) exhibited statistically significant upregulation in cancer tissues (*p *< 0.01). However, the expression levels of the two downregulated lncRNAs (lncRNA TCONS_00021144 and lncRNA TCONS_00282275) showed no significant difference between cancer and adjacent tissues (*p * > 0.05), as depicted in Figure 1a–e.

### 3.3 lncRNA TCONS_00251376 is Upregulated in GC Cell Lines

To assess whether the expression of lncRNA TCONS_00251376, lncRNA TCONS_00191782, and lncRNA TCONS_00184607 in GC cells surpasses that in normal gastric mucosal cells, we cultured normal gastric mucosal cells GES‐1 and GC cell lines AGS, MKN45, BGC‐823, and MGC‐803. The expression levels of lncRNA TCONS_00251376, lncRNA TCONS_00191782, and lncRNA TCONS_00184607 in GES‐1 and GC cell lines were determined through RNA extraction, reverse transcription, and qRT‐PCR. The results demonstrated that the expression level of lncRNA TCONS_00251376 in GC cells was significantly higher than that in normal gastric mucosa cell GES‐1, with MKN45 exhibiting the highest expression, followed by AGS. LncRNA TCONS_00191782 displayed significantly lower expression in GC cell lines AGS, MKN45, BGC‐823, and MGC‐803 compared to normal gastric mucosal cell GES‐1 (*p* < 0.001). However, there was no significant difference in the expression of lncRNA TCONS_00184607 between normal gastric mucosal cells and GC cells (*p* > 0.05), as illustrated in Figure 1f–h.

### 3.4 lncRNA TCONS_00251376 Interference Efficiency Detection

**Figure 2:**
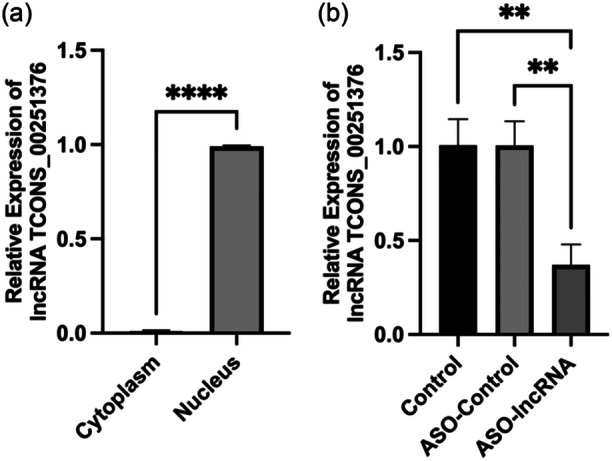
The expression of lncRNA TCONS_00251376 in AGS cells and ASO interfering availability. (a) The expression of lncRNA TCONS_00251376 in nucleus and cytoplasm of AGS cells. (b) The expression of lncRNA TCONS_00251376 in control group, ASO‐control group, and ASO‐lncRNA TCONS_00251376 group. **p < 0.01, ****p < 0.0001.

The expression and localization experiments of lncRNA TCONS_00251376 revealed predominant expression in the nucleus of AGS cells (Figure 2a). To investigate whether the expression of lncRNA TCONS_00251376 influences the biological function of GC cells, we employed the ASO method to silence lncRNA TCONS_00251376 in AGS cells, considering its relatively higher interference efficiency in the nucleus. The interference efficiency was subsequently assessed using qRT‐PCR. The experimental results indicated that compared with the control group and ASO‐control group, the expression of lncRNA TCONS_00251376 was significantly inhibited (*p *< 0.05), with an interference rate of approximately 63%, as depicted in Figure 2b.

### 3.5 lncRNA TCONS_00251376 Promotes GC Cell Proliferation, Migration, and Invasion In Vitro

**Figure 3:**
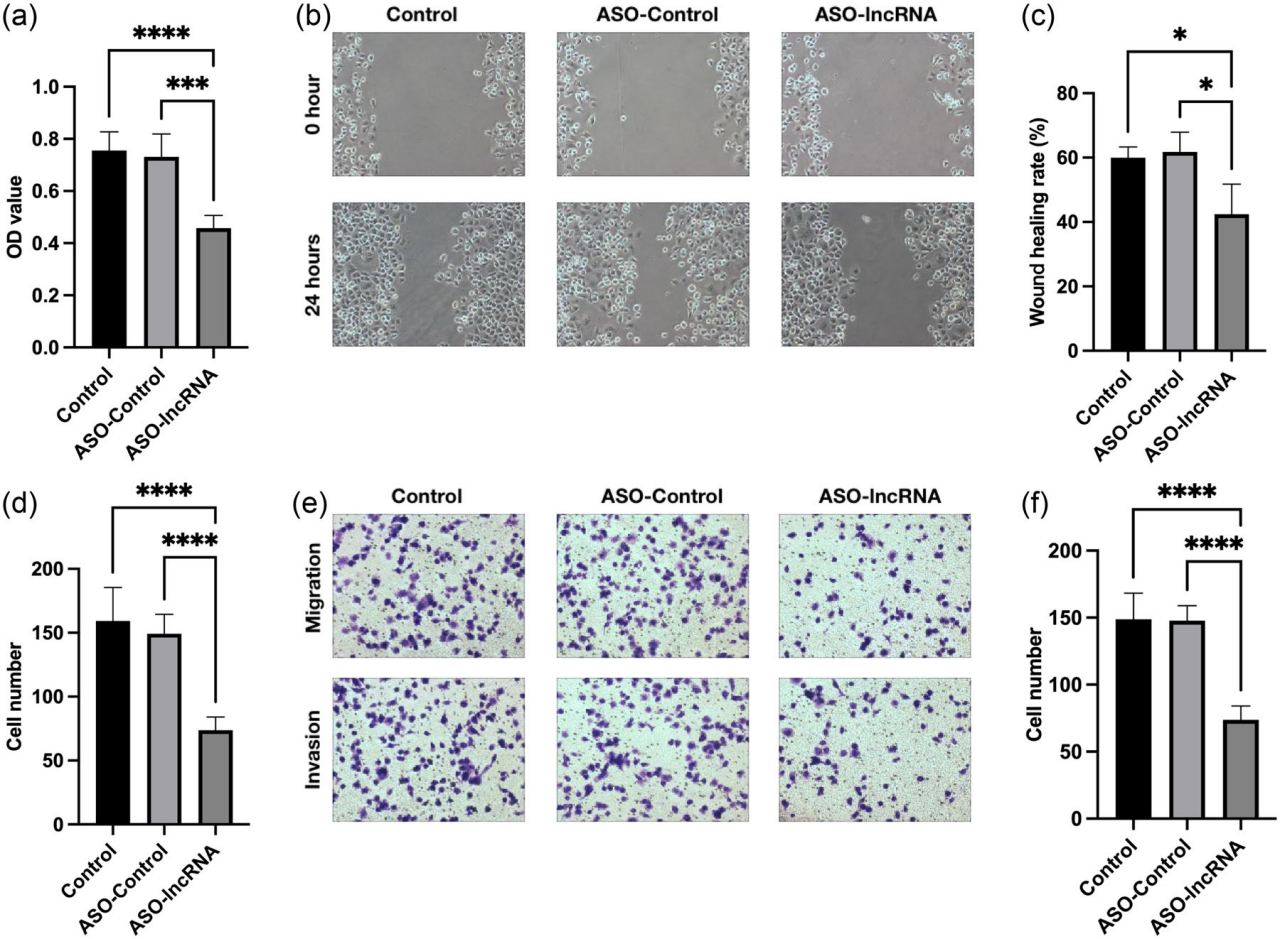
Influence of lncRNA TCONS_00251376 for cell proliferation, migration, and invasion of AGS. (a) Optical density value of AGS cells after 24‐h culture in CCK8 test. (b) Wound healing test of three groups of AGS cells. (c) Wound healing rates of three groups of AGS cells after 24 h culture. (d) Cell number of three groups of AGS cells after 24 h culture in cell migration test. (e) Cell migration and invasion test of three groups of AGS cells. (f) Cell number of three groups of AGS cells after 24 h culture in cell invasion test. *p < 0.05, ***p < 0.001, ****p < 0.0001.

To investigate the impact of lncRNA TCONS_00251376 silencing on the proliferation of GC cell AGS, we utilized the CCK8 method to assess the proliferation rate in the control group, ASO‐control group, and ASO‐lncRNA group of AGS. The results indicated that compared with the control group and the ASO‐control group, the ASO‐lncRNA group exhibited significantly lower absorbance (Optical density, OD) values after 24 h of culture (*p *< 0.05). This suggests that interference with lncRNA TCONS_00251376 leads to a significant decrease in the growth activity of AGS cells (Figure 3a), indicating that lncRNA TCONS_00251376 promotes the proliferation of AGS cells. Furthermore, Transwell chambers were employed to assess the migration and invasion abilities of the three groups of AGS cells. After 24 h of culture, the cells that traversed the chamber were stained and counted. The results revealed that in both migration and invasion experiments, the number of AGS cells passing through the chamber in the ASO‐lncRNA group was significantly lower than that in the control group and ASO‐control group. This indicates that lncRNA TCONS_00251376 promotes the migration and invasion of AGS GC cells (Figure 3b–f).

### 3.6 lncRNA TCONS_00251376 Promotes the Proliferation and Inhibits the Apoptosis of GC Cells

**Figure 4:**
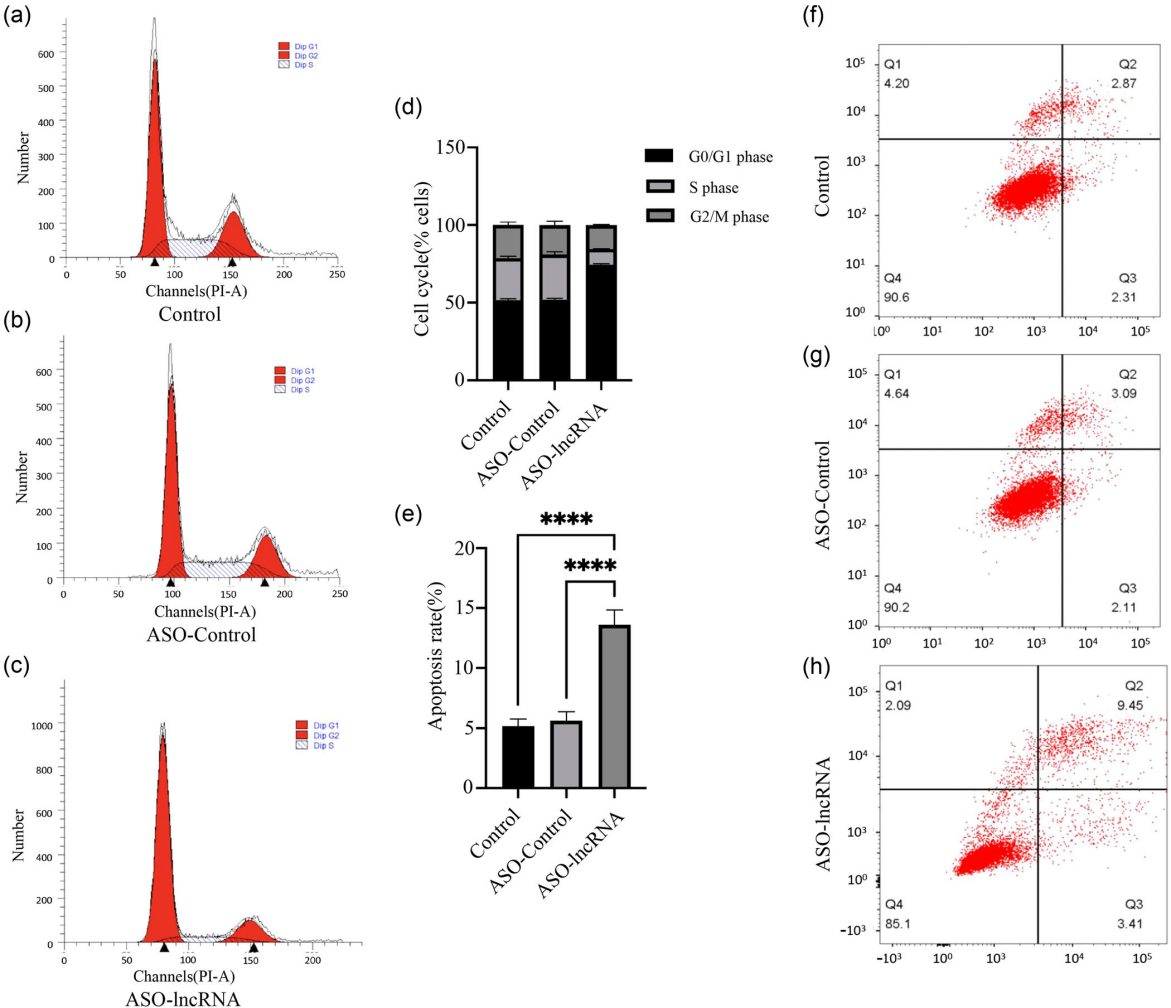
Influence of lncRNA TCONS_00251376 for cell cycle and apoptosis of AGS cell. (a–d) AGS cell distribution in cell cycle of different stage in Control, ASO‐control and ASO‐lncRNA group. (e–h) Apoptosis rate of AGS cells in Control, ASO‐control and ASO‐lncRNA group. ****p < 0.0001.

To examine the impact of silencing lncRNA TCONS_00251376 on the cell cycle of GC cells, flow cytometry was employed to measure the proportions of AGS cells in the G0/G1 phase, S phase, and G2/M phase in the three groups. The results demonstrated that compared with the control group and ASO‐control group, the proportion of AGS cells in the G0/G1 phase significantly increased in the ASO‐lncRNA group (74.5% *vs.* 51.6%, *p* < 0.05; 74.5% *vs*. 51.8%, *p* < 0.05), while the proportion of AGS cells in the S phase decreased significantly (10.1% *vs*. 27.1%, *p* < 0.05; 10.1% *vs*. 29.2%, *p *< 0.05). These findings indicate that lncRNA TCONS_00251376 promotes the growth of AGS GC cells (Figure 4a–d).

To investigate whether silencing lncRNA TCONS_00251376 can impact the apoptosis of GC cells, flow cytometry was employed to measure the apoptosis rate in three groups of AGS cells (control group, ASO‐control group, and ASO‐lncRNA group). The results revealed that compared with the control group and the ASO‐NC group, the apoptosis rate of the ASO‐lncRNA group significantly increased (13.6% vs. 5.2%, *p* < 0.05; 13.6% *vs*. 5.6%, *p *< 0.05). This indicates that lncRNA TCONS_00251376 can inhibit the apoptosis of GC cells (Figure 4e–h).

### 3.7 lncRNA TCONS_00251376 Can Upregulate ETV1 Gene and Influence Prognosis of GC

**Figure 5:**
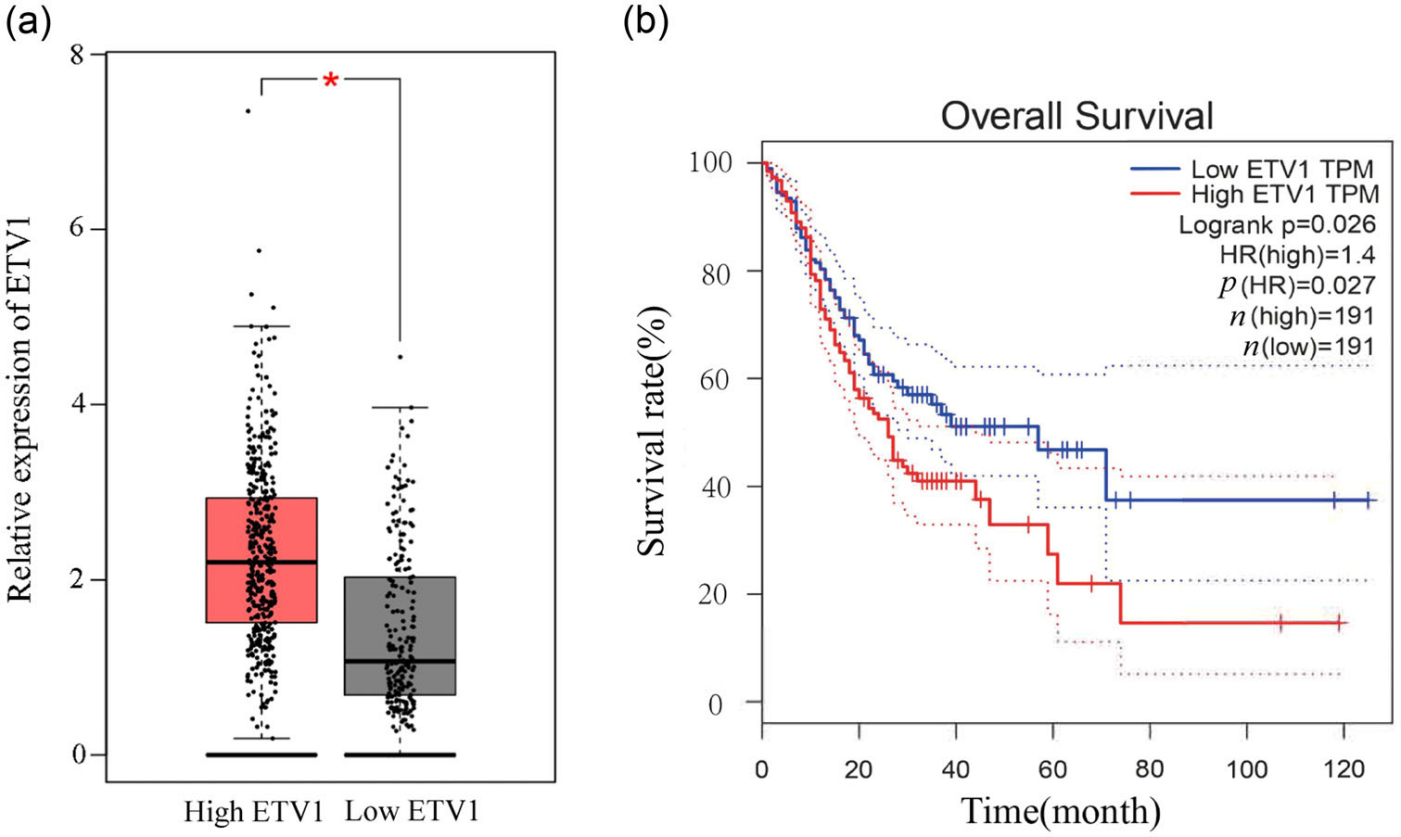
Expression and survival of ETV1 gene. (a) ETV1 gene was highly expressed in cancer than in paracancer tissue. (b) High expression of ETV1 gene indicated worse prognosis. *p < 0.05.

Upon inputting the sequence of lncRNA TCONS_00251376 into the Ensembl database (https://www.ensembl.org), we identified ETV1 as the downstream gene of lncRNA TCONS_00251376. A literature search revealed that ETV1 is closely related to gastric malignancy. Subsequently, we queried ETV1 in the gene expression comparison module of the GEPIA database. The results indicated that the expression of ETV1 in GC tissues was significantly higher than that in adjacent tissues (*p* < 0.01). Survival analysis further demonstrated that patients with high expression of ETV1 had a poorer prognosis (*p *= 0.026), as illustrated in Figure 5a,b.

**Figure 6:**
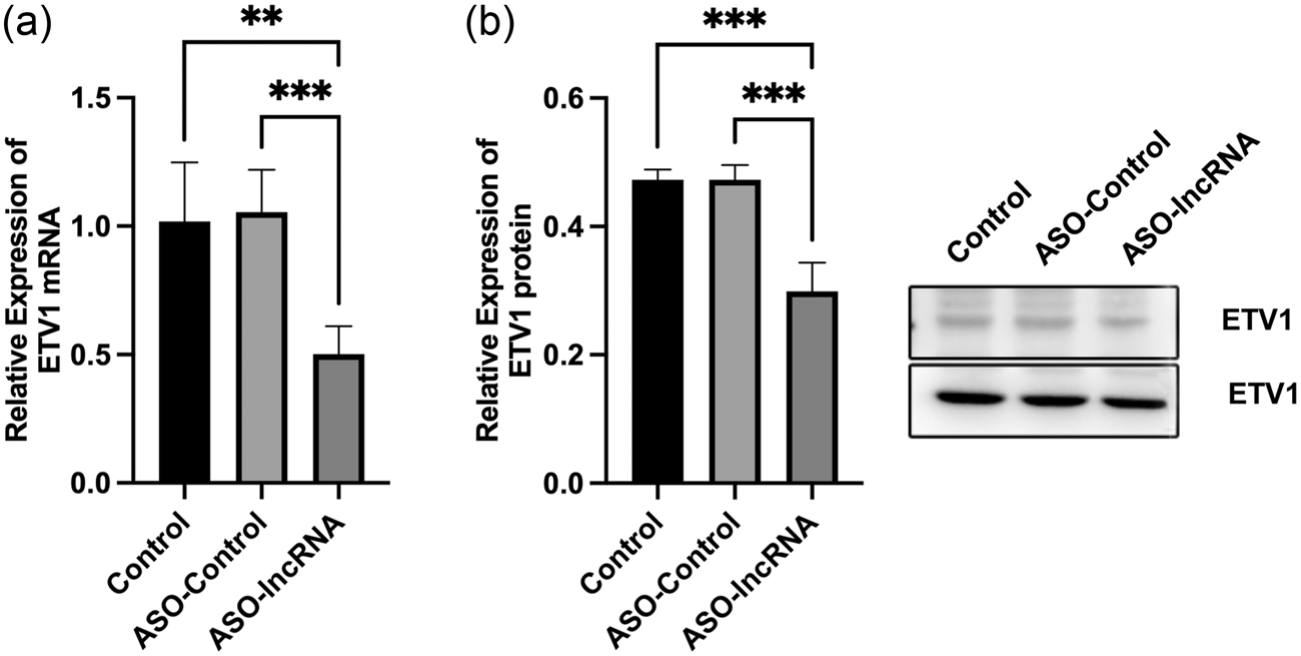
lncRNA TCONS_00251376 promoted the expression of ETV1 gene. (a) lncRNA TCONS_00251376 promoted the expression of ETV1 mRNA. (b) lncRNA TCONS_00251376 promoted the expression level of ETV1 protein. **p < 0.01, ***p < 0.001.

To further investigate whether lncRNA TCONS_00251376 can regulate the expression of ETV1 at the mRNA and protein levels, we assessed the changes in ETV1 mRNA and protein expression through qRT‐PCR and Western blot after interfering with lncRNA TCONS_00251376 in AGS GC cells. The results indicated that compared with the control and ASO‐NC group, the expression of ETV1 mRNA and protein was significantly reduced after silencing lncRNA TCONS_00251376 (*p *< 0.05). This is illustrated in Figure 6.

### 3.8 lncRNA TCONS_00251376 Promotes the Growth of AGS Tumor Cells In Vivo

**Figure 7:**
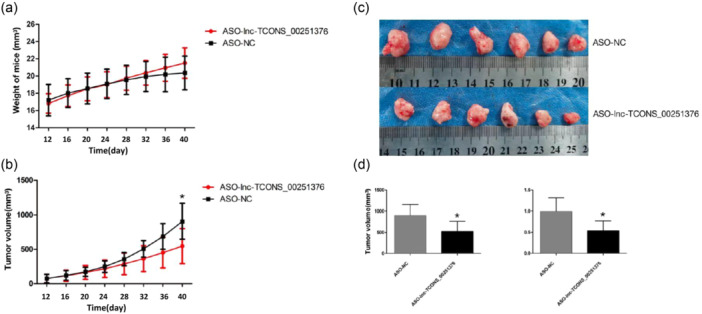
lncRNA TCONS_00251376 promotes GC cell in vivo. (a) Weight of mice in group ASO‐NC and group ASO‐lncRNA TCONS_00251376. (b) Tumor volume of two groups from Day 12 to Day 40. (c) Tumors excised from mice (*n* = 6). (d) Tumor volume of two groups when excised from mice.

To further investigate the role of lncRNA TCONS_00251376 in tumor proliferation, AGS cells were subcutaneously inoculated into the right forelimb armpit of BALB/c mice. Once the models were established successfully, ASO‐lnc‐TCONS_00251376 or ASO‐NC were injected multiple times intratumorally every 3 days. The results demonstrated that ASO‐lnc‐TCONS_00251376 significantly decreased tumor volumes compared to the control group (Figure 7), suggesting that lncRNA TCONS_00251376 promotes tumor proliferation.

## 4 Discussion

Noncoding RNAs (ncRNAs) constitute an emerging class of transcripts that were initially thought to lack the potential for protein encoding. However, it has been discovered that more than 80% of the entire human genome can be transcribed into ncRNAs [8]. Certain ncRNAs play pivotal roles in various diseases, including GC [9]. Based on their length, ncRNAs are categorized into lncRNAs and small ncRNAs [10]. Initially perceived as transcriptional “garbage” or “noise” of genes, recent studies have increasingly highlighted the crucial role of lncRNAs in physiological and pathological processes, encompassing gene expression regulation, embryonic development, and carcinogenesis [11, 12]. Currently, dysregulated expression of lncRNAs has been observed in numerous malignancies, including colon cancer, breast cancer, prostate cancer, and GC [13, 14, 15, 16].

In recent years, the rapid advancement of RNA next‐generation sequencing technology has led to the discovery of numerous lncRNAs in GC. Investigating the mechanisms of these lncRNAs holds significant promise for enhancing the diagnosis and treatment of GC. LncRNA TCONS_00251376, identified through RNA next‐generation sequencing, is a novel lncRNA. Increasing evidence from studies indicates that lncRNAs play crucial roles in the progression of GC by targeting signaling pathways, such as PI3K/AKT, mitogen‐activated protein kinase (MAPK), Wnt/β‐catenin, and STAT3 signal pathways [17, 18, 19, 20, 21]. The PI3K/AKT signaling pathway is known to regulate various functions of GC cells, including proliferation, metastasis, and drug resistance. Dysregulation of the PI3K/AKT signaling pathway is closely associated with the progression of GC [17]. Multiple studies have demonstrated that lncRNAs act as key regulators of the PI3K/AKT signaling pathway. For instance, Shi et al. observed that overexpression of lncRNA LOC101928316 in the GC cell line SGC‐7901 significantly inhibited the expression of PI3K, p‐AKT, mTOR, and p‐mTOR, suggesting the involvement of lncRNA LOC101928316 in GC progression by inhibiting the PI3K/AKT signaling pathway [22]. Additionally, several other lncRNAs, such as XLOC_006753, OGFRP1, TMPO‐AS1, and FOXD1‐AS1, have been reported to participate in the regulation of the PI3K/AKT signaling pathway in GC [23, 24, 25, 26]. Furthermore, the MAPK pathway, closely linked to fundamental cellular functions like proliferation, apoptosis, migration, and senescence, has been found to be dysregulated in malignant tumors, including GC [18, 19]. Certain lncRNAs, including LINC00152‐1, CASC2, and AK025387, play significant roles in the progression of GC by modulating the MAPK pathway [22, 27, 28].

Our study suggests that lncRNA TCONS_00251376 may function as an oncogene in the initiation and progression of GC. We examined its expression in 22 pairs of gastric adenocarcinoma tissues and adjacent tissues, revealing significantly higher expression in cancer tissues compared to adjacent tissues. Cellular experiments further confirmed elevated expression of lncRNA TCONS_00251376 in four GC cell lines (AGS, MKN45, BGC‐823, and MGC‐803) compared to normal gastric mucosal epithelial cells (GES‐1). Studies on lncRNAs in GC consistently show that many GC‐related lncRNAs are highly expressed in cancer tissues and associated with cancer progression. For instance, ZFAS1 exhibits high expression in GC tumor tissues, promoting the migration and invasion of GC cells by regulating epithelial‐mesenchymal transition [29]. HOTAIR, the first lncRNA confirmed to play a crucial role in tumor invasion and metastasis, also shows significantly higher expression in GC tissues compared to adjacent tissues [30]. Moreover, certain lncRNAs are highly expressed not only in GC tissues but also in serum, gastric juice, and other samples from GC patients. RMRP and H19, for instance, are highly expressed in both GC tissues and gastric juice, with RMRP levels correlating with the pathological type and metastasis of GC [31]. H19 promotes epithelial‐mesenchymal transition, enhancing the invasion and metastasis abilities of GC cells by downregulating E‐cadherin expression [32]. However, some studies [33, 34] have identified a subset of lncRNAs that are lowly expressed in GC tissues and play important roles in cancer initiation and development, often acting as tumor suppressors. MEG3, expressed in normal gastric mucosal epithelial tissues but not in GC tissues, inhibits the proliferation and invasion abilities of GC cells when overexpressed [33]. GAS5, downregulated in GC tissues compared to adjacent tissues, is associated with the apoptosis of GC cells [34]. Additionally, other lncRNAs have been identified that suppress the proliferation and invasion of GC cells and are downregulated in GC tissues [35, 36].

Numerous studies have consistently demonstrated that lncRNAs are predominantly expressed in the nucleus. Our subcellular localization experiment for lncRNA TCONS_00251376 in this study also confirmed its predominant expression in the nucleus. To delve into the biological function of lncRNA TCONS_00251376, we selected the normal gastric mucosal epithelial cell line GES‐1 and the GC cell line AGS, characterized by relatively high lncRNA TCONS_00251376 expression and adherent growth, for further cytological investigations. Employing the ASO method, we successfully interfered with lncRNA TCONS_00251376. Upon successful interference, we observed a decrease in the proliferation rate of GC cells (AGS), a significant increase in the proportion of cells in the G0/G1 phase, and a reduction in the migration and invasion abilities of AGS cells. These findings suggest that lncRNA TCONS_00251376 plays a role in promoting the proliferation of GC cells, enhancing their migration and invasion capabilities. Furthermore, our animal experiment indicated that the knockdown of lncRNA TCONS_00251376 suppressed tumor proliferation. This observed effect aligns with the trends observed in many previous studies involving highly expressed lncRNAs in GC tissues.

Furthermore, we also explored the possible mechanism of the cancer‐promoting effect of lncRNA TCONS_00251376. We inputted the sequence of lncRNA TCONS_00251376 into the Ensembl database and found that there were two downstream genes nearby—ETV1 and DGKB. ETV1 is a member of the ETS transcription factor family, and ETS protein can regulate a variety of genes that adjust biological processes, such as those related to cell growth, migration, proliferation, and differentiation [37]. PEA3 is a subfamily of the ETS transcription factor family, and ETV1 is a member of the PEA3 subfamily. The PEA3 subfamily also includes ETV4, ETV5, and other genes. Studies have shown that ETV1, ETV4, and ETV5 are highly expressed in a variety of cancers. ETV1, ETV4, and ETV5 can enhance the proliferation, migration, and invasion of tumor cells, thereby leading to tumor progression, metastasis, and drug resistance [38]. ETV1 is located on chromosome 7, and its protein structure is 95% identical to ETV4 and ETV5 proteins in the DNA binding domain [39]. Studies have found that ETV1 has the highest expression in prostate cancer [39, 40] and has the most specific expression in gastrointestinal stromal tumor [41]; moreover, its expression in gastric cancer is also significantly increased [42]. At present, there are lots of studies on ETV1 in prostate cancer. ETV1 can stabilize β‐catenin, which leads to increased accumulation of β‐catenin in prostate cancer cells and promotes malignant transformation of tumors. ETV1 can activate its target genes MMP‐1 and MMP‐7 to regulate cell migration and invasion [39]. In addition, ETV1 promotes prostate tumor progression by directly inhibiting CHK1 expression [40]. Moreover, ETV1 also plays a very important role in breast and pancreatic cancer. In breast cancer, HER2 interacts with ETV1 to synergistically activate the transcription of hTERT, making breast cancer more invasive; ETV1 can also coordinate with HER2/Neu to upregulate the expression of Rcl, and the combination of ETV1 and the Rcl is related with later tumor staging [43]. In pancreatic cancer, ETV1 increases the invasive ability of pancreatic cancer cells by regulating two downstream genes Sparc and Has2 [44].

Upon further literature review, it was revealed that ETV1 is highly expressed in GC tissues and exerts a promoting effect on the epithelial‐mesenchymal transition (EMT) of GC cells. A study conducted by Jang et al. [42], comparing the expression of ETV1 in 39 GC tissues and 15 normal gastric mucosal tissues, demonstrated significantly higher ETV1 expression in GC tissues. This finding was corroborated by a broader analysis of 408 GC tissues and 211 gastric mucosal tissues in the TCGA and GTEx databases, revealing elevated ETV1 expression in GC tissues compared to normal gastric mucosal tissues (*p* < 0.01). Furthermore, patients with high ETV1 expression in GC tissues exhibited significantly worse overall survival than those with low ETV1 expression (*p* = 0.026). In an in vitro study conducted by Keld et al. [45], the expression of ETV1 in GC cell lines MKN‐45, MGC‐803, and SGC‐7901 was found to be significantly higher than that in normal gastric mucosal cell GES‐1 at both mRNA and protein levels. The study demonstrated that overexpression of ETV1 in normal gastric mucosal cells induced EMT and increased their invasive ability. Conversely, knockdown of ETV1 in SGC‐7901 cells significantly reduced their invasive ability. The study further revealed that ETV1 induced EMT in gastric mucosal cells by upregulating the expression of the zinc finger transcription factor Snail, thereby promoting GC metastasis. In line with these findings, the present study indicated that silencing lncRNA TCONS_00251376 significantly reduced the expression of ETV1 at both mRNA and protein levels. This suggests that lncRNA TCONS_00251376 might promote the proliferation, migration, and invasion of tumor cells by upregulating the expression of ETV1. The identified relationship between lncRNA TCONS_00251376 and ETV1 adds to the understanding of potential molecular mechanisms underlying GC progression.

## 5 Conclusions

Our study provides evidence that lncRNA TCONS_00251376 functions as an oncogenic lncRNA, promoting tumorigenesis and progression by regulating the expression of ETV1 gene. These findings underscore the potential of lncRNA TCONS_00251376 as a promising therapeutic target for the treatment of GC.

## Author Contributions

**Dengfeng Ren:** Data curation (equal), writing – original draft (equal). **Fuxing Zhao:** Data curation (equal), writing – original draft (equal). **Jinming Li:** Data curation (equal), writing – original draft (equal). **Xinjian Guo:** Formal analysis (equal). **Xinfu Ma:** Conceptualization (equal), methodology (equal). **Yonghui Zheng:** Data curation (supporting), software (supporting). **Guoshuang Shen:** Conceptualization (lead), project administration (lead). **Jiuda Zhao:** Conceptualization (lead), project administration (lead).

## Ethics Statement

This study was conducted in accordance with the Declaration of Helsinki and approved by the Institutional Research Ethics Committee of Affiliated Hospital of Qinghai University (approval number: P‐SL‐2019041).

## Consent

Informed consent was obtained from all subjects involved in this study.

## Conflicts of Interest

Professor Jiuda Zhao is a member of the *Cancer Innovation* Editorial Board. To minimize bias, he was excluded from all editorial decision‐making related to the acceptance of this article for publication. The remaining authors declare no conflicts of interest.

## Data Availability

The data used in the work is all open source data on the web.

## References

[1] H. Sung , J. Ferlay , R. L. Siegel , et al., “Global Cancer Statistics 2020: Globocan Estimates of Incidence and Mortality Worldwide for 36 Cancers in 185 Countries,” CA: A Cancer Journal for Clinicians 71, no. 3 (2021): 209–249, 10.3322/caac.21660. 33538338

[2] W. Cao , H. D. Chen , Y. W. Yu , N. Li , and W. Q. Chen , “Changing Profiles of Cancer Burden Worldwide and in China: A Secondary Analysis of the Global Cancer Statistics 2020,” Chinese Medical Journal 134, no. 7 (2021): 783–791, 10.1097/CM9.0000000000001474. 33734139 PMC8104205

[3] L. Zong , M. Abe , Y. Seto , and J. Ji , “The Challenge of Screening for Early Gastric Cancer in China,” Lancet 388, no. 10060 (2016): 2606, 10.1016/S0140-6736(16)32226-7. 27894662

[4] L. Shen , Y. S. Shan , H. M. Hu , et al., “Management of Gastric Cancer in Asia: Resource‐Stratified Guidelines,” Lancet Oncology 14, no. 12 (2013): e535–e547, 10.1016/S1470-2045(13)70436-4. 24176572

[5] E. Van Cutsem , X. Sagaert , B. Topal , K. Haustermans , and H. Prenen , “Gastric Cancer,” Lancet 388, no. 10060 (2016): 2654–2664, 10.1016/S0140-6736(16)30354-3. 27156933

[6] F. Lordick and Y. Y. Janjigian , “Clinical Impact of Tumour Biology in the Management of Gastroesophageal Cancer,” Nature Reviews Clinical Oncology 13, no. 6 (2016): 348–360, 10.1038/nrclinonc.2016.15. PMC5521012 26925958

[7] K. A. Lennox and M. A. Behlke , “Cellular Localization of Long Non‐Coding RNAs Affects Silencing by RNAi More Than by Antisense Oligonucleotides,” Nucleic Acids Research 44, no. 2 (2016): 863–877, 10.1093/nar/gkv1206. 26578588 PMC4737147

[8] E. S. Martens‐Uzunova , R. Böttcher , C. M. Croce , G. Jenster , T. Visakorpi , and G. A. Calin , “Long Noncoding RNA in Prostate, Bladder, and Kidney Cancer,” European Urology 65, no. 6 (2014): 1140–1151, 10.1016/j.eururo.2013.12.003. 24373479

[9] M. Zhao , N. Zhu , F. Hao , et al., “The Regulatory Role of Non‐Coding Rnas on Programmed Cell Death Four in Inflammation and Cancer,” Frontiers in Oncology 9 (2019): 919, 10.3389/fonc.2019.00919. 31620370 PMC6759660

[10] P. Svoboda , “Long and Small Noncoding RNAs During Oocyte‐to‐Embryo Transition in Mammals,” Biochemical Society Transactions 45, no. 5 (2017): 1117–1124, 10.1042/BST20170033. 28939692

[11] M. T. Lin , H. J. Song , and X. Y. Ding , “Long Non‐Coding RNAs Involved in Metastasis of Gastric Cancer,” World Journal of Gastroenterology 24, no. 33 (2018): 3724–3737, 10.3748/wjg.v24.i33.3724. 30197478 PMC6127659

[12] W. Sun , Y. Yang , C. Xu , and J. Guo , “Regulatory Mechanisms of Long Noncoding RNAs on Gene Expression in Cancers,” Cancer Genetics 216–217 (2017): 105–110, 10.1016/j.cancergen.2017.06.003. 29025584

[13] R. Begolli , N. Sideris , and A. Giakountis , “Lncrnas as Chromatin Regulators in Cancer: From Molecular Function to Clinical Potential,” Cancers 11, no. 10 (2019): 1524, 10.3390/cancers11101524. 31658672 PMC6826483

[14] Y. Li , X. Liu , X. Cui , et al., “LncRNA Pradx‐Mediated Recruitment of PRC2/DDX5 Complex Suppresses UBXN1 Expression and Activates NF‐κB Activity, Promoting Tumorigenesis,” Theranostics 11, no. 9 (2021): 4516–4530, 10.7150/thno.54549. 33754075 PMC7977445

[15] P. Zhu , F. He , Y. Hou , et al., “A Novel Hypoxic Long Noncoding RNA KB‐1980E6.3 Maintains Breast Cancer Stem Cell Stemness *via* Interacting With IGF_2_BP_1_ to Facilitate c‐Myc mRNA stability,” Oncogene 40 (2021): 1609–1627, 10.1038/s41388-020-01638-9. 33469161 PMC7932928

[16] F. Zhang , H. Wang , J. Yu , et al., “LncRNA CRNDE Attenuates Chemoresistance in Gastric Cancer *via* SRSF6‐Regulated Alternative Splicing of PICALM,” Molecular Cancer 20, no. 1 (2021): 6, 10.1186/s12943-020-01299-y. 33397371 PMC7780690

[17] Q. Dai , T. Zhang , and C. Li , “LncRNA MALAT1 Regulates the Cell Proliferation and Cisplatin Resistance in Gastric Cancer via PI3K/AKT Pathway,” Cancer Management and Research 12 (2020): 1929–1939, 10.2147/CMAR.S243796. 32214850 PMC7078812

[18] Y. J. Guo , W. W. Pan , S. B. Liu , Z. F. Shen , Y. Xu , and L. L. Hu , “ERK/MAPK Signalling Pathway and Tumorigenesis,” Experimental and Therapeutic Medicine 19, no. 3 (2020): 1997–2007, 10.3892/etm.2020.8454. 32104259 PMC7027163

[19] M. Yang , “Mitogen‐Activated Protein Kinase Signaling Pathway and Invasion and Metastasis of Gastric Cancer,” World Journal of Gastroenterology 21, no. 41 (2015): 11673–11679, 10.3748/wjg.v21.i41.11673. 26556994 PMC4631968

[20] H. Zhang , H. Huang , X. Xu , et al., “LncRNA HCG11 Promotes Proliferation and Migration in Gastric Cancer *via* Targeting miR‐1276/CTNNB1 and Activating Wnt Signaling Pathway,” Cancer Cell International 19 (2019): 350, 10.1186/s12935-019-1046-0. 31889902 PMC6933929

[21] M. Ashrafizadeh , A. Zarrabi , S. Orouei , et al., “STAT3 Pathway in Gastric Cancer: Signaling, Therapeutic Targeting and Future Prospects,” Biology 9, no. 6 (2020): 126, 10.3390/biology9060126. 32545648 PMC7345582

[22] Y. Shi and H. Sun , “Down‐Regulation of LncRNA LINC00152 Suppresses Gastric Cancer Cell Migration and Invasion Through Inhibition of the ERK/MAPK Signaling Pathway,” OncoTargets and Therapy 13 (2020): 2115–2124, 10.2147/OTT.S217452. 32210577 PMC7074822

[23] L. Zeng , Q. Liao , Z. Zou , et al., “Long Non‐Coding RNA XLOC_006753 Promotes the Development of Multidrug Resistance in Gastric Cancer Cells Through the PI3K/AKT/mTOR Signaling Pathway,” Cellular Physiology and Biochemistry 51, no. 3 (2018): 1221–1236, 10.1159/000495499. 30481766

[24] J. Zhang , X. Xu , J. Yin , et al., “LncRNA OGFRP1 Promotes Tumor Progression By Activating the AKT/mTOR Pathway in Human Gastric Cancer,” Aging 13, no. 7 (2021): 9766–9779, 10.18632/aging.202731. 33744848 PMC8064230

[25] Q. Wu , J. Ma , J. Wei , W. Meng , Y. Wang , and M. Shi , “FOXD1‐AS1 Regulates FOXD1 Translation and Promotes Gastric Cancer Progression and Chemoresistance by Activating the PI3K/AKT/mTOR Pathway,” Molecular Oncology 15, no. 1 (2021): 299–316, 10.1002/1878-0261.12728. 32460412 PMC7782086

[26] Y. Hu , Y. Zhang , M. Ding , and R. Xu , “Long Noncoding RNA TMPO‐AS1/miR‐126‐5p/BRCC3 Axis Accelerates Gastric Cancer Progression and Angiogenesis *via* Activating PI3K/Akt/mTOR pathway,” Journal of Gastroenterology and Hepatology 36, no. 7 (2021): 1877–1888, 10.1111/jgh.15362. 33295056

[27] P. Li , W. J. Xue , Y. Feng , and Q. S. Mao , “Long Non‐Coding RNA CASC2 Suppresses the Proliferation of Gastric Cancer Cells by Regulating the MAPK Signaling Pathway,” American Journal of Translational Research 8, no. 8 (2016): 3522–3529. 27648142 PMC5009404

[28] Y. Y. Sun , H. Zhang , R. R. Ma , et al., “Long Non‐Coding RNA AK025387 Promotes Cell Migration and Invasion of Gastric Cancer,” Frontiers in Oncology 10 (2020): 633, 10.3389/fonc.2020.00633. 32509569 PMC7251172

[29] L. Pan , W. Liang , M. Fu , et al., “Exosomes‐Mediated Transfer of Long Noncoding RNA ZFAS1 Promotes Gastric Cancer Progression,” Journal of Cancer Research and Clinical Oncology 143, no. 6 (2017): 991–1004, 10.1007/s00432-017-2361-2. 28285404 PMC11819301

[30] Z. Wei , L. Chen , L. Meng , W. Han , L. Huang , and A. Xu , “LncRNA Hotair Promotes the Growth and Metastasis of Gastric Cancer by Sponging miR‐1277‐5p and Upregulating COL5A1,” Gastric Cancer 23, no. 6 (2020): 1018–1032, 10.1007/s10120-020-01091-3. 32583079

[31] H. L. Cao , Z. J. Liu , P. L. Huang , Y. L. Yue , and J. N. Xi , “lncRNA‐RMRP Promotes Proliferation, Migration and Invasion of Bladder Cancer *via* miR‐206,” European Review for Medical and Pharmacological Sciences 23, no. 3 (2019): 1012–1021, 10.26355/eurrev_201902_16988. 30779067

[32] G. Liu , T. Xiang , Q. F. Wu , and W. X. Wang , “Long Noncoding RNA H19‐Derived miR‐675 Enhances Proliferation and Invasion *via* RUNX1 in Gastric Cancer Cells,” Oncology Research Featuring Preclinical and Clinical Cancer Therapeutics 23, no. 3 (2016): 99–107, 10.3727/096504015X14496932933575. PMC7838630 26931432

[33] J. Dan , J. Wang , Y. Wang , et al., “LncRNA‐MEG3 Inhibits Proliferation and Metastasis by Regulating miRNA‐21 in Gastric Cancer,” Biomedicine & Pharmacotherapy 99 (2018): 931–938, 10.1016/j.biopha.2018.01.164. 29710493

[34] N. Zhang , A. Y. Wang , X. K. Wang , X. M. Sun , and H. Z. Xue , “GAS5 Is Downregulated in Gastric Cancer Cells by Promoter Hypermethylation and Regulates Adriamycin Sensitivity,” European Review for Medical and Pharmacological Sciences 20, no. 15 (2016): 3199–3205. 27466992

[35] T. Xu , M. Huang , R. Xia , et al., “Decreased Expression of the Long Non‐Coding RNA FENDRR Is Associated With Poor Prognosis in Gastric Cancer and FENDRR Regulates Gastric Cancer Cell Metastasis by Affecting fibronectin1 Expression,” Journal of Hematology & Oncology 7 (2014): 63, 10.1186/s13045-014-0063-7. 25167886 PMC4237812

[36] L. Liu , B. Yan , Z. Yang , X. Zhang , Q. Gu , and X. Yue , “Ncrupar Inhibits Gastric Cancer Progression by Down‐Regulating Protease‐Activated Receptor‐1,” Tumor Biology 35, no. 8 (2014): 7821–7829, 10.1007/s13277-014-2042-6. 24817013

[37] T. Oikawa , “ETS Transcription Factors: Possible Targets for Cancer Therapy,” Cancer science 95, no. 8 (2004): 626–633, 10.1111/j.1349-7006.2004.tb03320.x. 15298723 PMC11159856

[38] T. Qi , Q. Qu , G. Li , et al., “Function and Regulation of the PEA3 Subfamily of ETS Transcription Factors in Cancer,” American Journal of Cancer Research 10, no. 10 (2020): 3083–3105. 33163259 PMC7642666

[39] A. C. Rodriguez , J. M. Vahrenkamp , K. C. Berrett , et al., “ETV4 Is Necessary for Estrogen Signaling and Growth in Endometrial Cancer Cells,” Cancer Research 80, no. 6 (2020): 1234–1245, 10.1158/0008-5472.CAN-19-1382. 32046982 PMC7073291

[40] S. Morsalin , C. Yang , J. Fang , et al., “Molecular Mechanism of *β*‐Catenin Signaling Pathway Inactivation in ETV1‐Positive Prostate Cancers,” Journal of Pharmaceutical Sciences and Pharmacology 2, no. 3 (2015): 208–216, 10.1166/jpsp.2015.1069. 28497076 PMC5423671

[41] W. Eid and W. Abdel‐Rehim , “Genome‐Wide Analysis of ETV1 Targets: Insights Into the Role of ETV1 in Tumor Progression,” Journal of Cellular Biochemistry 120, no. 6 (2019): 8983–8991, 10.1002/jcb.28169. 30629294

[42] B. G. Jang , H. E. Lee , and W. H. Kim , “ETV1 mRNA Is Specifically Expressed in Gastrointestinal Stromal Tumors,” Virchows Archiv 467, no. 4 (2015): 393–403, 10.1007/s00428-015-1813-9. 26243012

[43] S. Shin , D. G. Bosc , J. N. Ingle , T. C. Spelsberg , and R. Janknecht , “Rcl Is a Novel *ETV1*/*ER81* Target Gene Upregulated in Breast Tumors,” Journal of Cellular Biochemistry 105, no. 3 (2008): 866–874, 10.1002/jcb.21884. 18726892

[44] S. Heeg , K. K. Das , M. Reichert , et al., “Ets‐Transcription Factor ETV1 Regulates Stromal Expansion and Metastasis in Pancreatic Cancer,” Gastroenterology 151, no. 3 (2016): 540–553.e14, 10.1053/j.gastro.2016.06.005. 27318148 PMC5002361

[45] R. Keld , B. Guo , P. Downey , et al., “PEA3/ETV4‐Related Transcription Factors Coupled With Active ERK Signalling Are Associated With Poor Prognosis in Gastric Adenocarcinoma,” British Journal of Cancer 105, no. 1 (2011): 124–130, 10.1038/bjc.2011.187. 21673681 PMC3137405

